# Clinical TNM staging for esophageal, gastric, and colorectal cancers in the era of neoadjuvant therapy: A systematic review of the literature

**DOI:** 10.1002/ags3.12444

**Published:** 2021-02-18

**Authors:** Hideaki Shimada, Takeo Fukagawa, Yoshio Haga, Shin‐ichi Okazumi, Koji Oba

**Affiliations:** ^1^ Department of Gastroenterological Surgery Toho University Graduate School of Medicine Tokyo Japan; ^2^ Department of Surgery Teikyo University School of Medicine Tokyo Japan; ^3^ Department of Surgery Japan Community Healthcare Organization Amakusa Central General Hospital Amakusa Japan; ^4^ Department of Surgery Toho University Sakura Medical Center Sakura Japan; ^5^ Department of Biostatistics School of Public Health Graduate School of Medicine The University of Tokyo Tokyo Japan; ^6^ Interfaculty Initiative in Information Studies Graduate School of Interdisciplinary Information Studies The University of Tokyo Tokyo Japan

**Keywords:** colorectal cancer, distant metastases, esophageal cancer, gastric cancer, lymph node metastases, T4

## Abstract

**Aim:**

Clinical staging is vital for selecting appropriate candidates and designing neoadjuvant treatment strategies for advanced tumors. The aim of this review was to evaluate diagnostic abilities of clinical TNM staging for gastrointestinal, gastrointestinal cancers.

**Methods:**

We conducted a systematic review of recent publications to evaluate the accuracy of diagnostic modalities on gastrointestinal cancers. A systematic literature search was performed in PubMed/MEDLINE using the keywords “TNM staging,” “T4 staging,” “distant metastases,” “esophageal cancer,” “gastric cancer,” and “colorectal cancer,” and the search terms used in Cochrane Reviews between January 2005 to July 2020. Articles focusing on preoperative diagnosis of: (a) depth of invasion; (b) lymph node metastases; and (c) distant metastases were selected.

**Results:**

After a full‐text search, a final set of 55 studies (17 esophageal cancer studies, 26 gastric cancer studies, and 12 colorectal cancer studies) were used to evaluate the accuracy of clinical TNM staging. Positron emission tomography–computed tomography (PET‐CT) and/or magnetic resonance imaging (MRI) were the best modalities to assess distant metastases. Fat and fiber mode of CT may be useful for T4 staging of esophageal cancer, CT was a partially reliable modality for lymph node staging in gastric cancer, and CT combined with MRI was the most reliable modality for liver metastases from colorectal cancer.

**Conclusion:**

The most reliable diagnostic modality differed among gastrointestinal cancers depending on the type of cancer. Therefore, we propose diagnostic algorithms for clinical staging for each type of cancer.

## Graphical abstract

We conducted a systematic review of recent publications to evaluate the accuracy of diagnostic modalities on gastrointestinal cancers. Such systematic review on all three cancer types—esophageal cancer, gastric cancer, colorectal cancer—cannot be found in recent publications on PubMed. We believe that this review may be fundamental information to design clinical studies related to neoadjuvant therapy for gastrointestinal cancers.

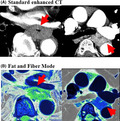

## 1 INTRODUCTION

Despite recent advances in surgical techniques, perioperative care, and multimodal treatment, postoperative recurrence is observed in approximately 40% of esophageal cancers^1^ and 20%‐30% of gastric^2^ and colorectal^3^ cancers with advanced tumors.^4^, ^5^, ^6^ Lymph node metastasis remains crucial for applying adjuvant treatment and predicting oncological outcome. Various studies have shown that such postoperative recurrence was frequently reduced by neoadjuvant chemotherapy (NAC).^7^, ^8^, ^9^, ^10^, ^11^ The present review evaluates the accuracy of preoperative diagnosis in gastrointestinal cancers, including lymph node metastasis.

Potential T4 esophageal cancer should be treated with neoadjuvant chemoradiation therapy to ensure a negative surgical margin for cancer cells. NAC became the standard management for stage II/III esophageal cancer following the results of the JCOG9907 trial.^12^ JCOG1002 evaluated NAC for locally advanced gastric cancer with extended lymph node metastasis and/or bulky positive nodes.^13^ Two more ongoing trials also evaluating NAC for locally advanced gastric cancer.^14^, ^15^ Distant metastases should be classified as a noncurative factor for surgical approach. Therefore, clinical TNM staging should be accurate, based on high sensitivity and specificity to predict T4 and/or distant metastases. Since definitive chemoradiation therapy showed a similar overall survival to radical surgery for clinical stage I esophageal cancer,^16^ an accurate diagnosis of lymph node metastases is also vital to design treatment strategies for potential stage I tumors.

In Western countries, preoperative chemotherapy or chemoradiotherapy is a standard therapeutic strategy for advanced gastric cancer, based on the findings from large‐scale randomized clinical trials.^17^, ^18^, ^19^, ^20^ While advanced stage gastric cancer is the main target of NAC, 8.3% of pathological T1 patients were included in the surgery alone group,^17^ indicating that some early gastric cancer patients underwent unnecessary NAC. This problem may be due to inaccuracy of clinical diagnosis of T and N staging. In Japan, the efficacy of NAC for type 4 and large‐sized type 3 was not demonstrated in the JCOG0501 trial.^21^ The JCOG1302A trial, which evaluated the accuracy of clinical diagnosis of gastric cancer, was conducted as prospective setting prior to starting the JCOG1509 trial^22^ regarding the efficacy of NAC for stage III gastric cancer.^23^

The JCOG1310 trial (PRECIOUS study) is intended to compare preoperative vs postoperative chemotherapy for lower rectal cancer patients with suspected lateral pelvic node metastasis.^24^ MRI has been reported to be the most effective tool for the preoperative stage diagnosis of rectal cancer.^25^ It remains controversial whether chemotherapy with or without primary tumor resection is effective for patients with incurable stage IV colorectal cancer. The precise detection of distant metastases^26^ is vital in order to enroll patients for such a typical randomized study.

Thus, the impact of clinical TNM staging is more important than ever since neoadjuvant therapy for gastrointestinal cancers is becoming established. Therefore, we evaluated the accuracy of clinical TNM staging through multimodal diagnostic tools using a systematic review of recent publications from January 2005 to July 2020. We propose the use of standard diagnostic algorithms for gastrointestinal cancers. The present review aimed to summarize the fundamental information about the accuracy of clinical TNM staging to design future guidelines and clinical protocols for preoperative adjuvant therapy for gastrointestinal cancers.

## 2 METHODS

### 2.1 Research themes and study selection criteria

The present review focused on esophageal, gastric, and colorectal cancers. An eligible trial was a clinical study which evaluate accuracy of clinical TNM staging based on imaging modalities including computed tomography (CT), magnetic resonance imaging (MRI), and positron emission tomography–computed tomography (PET‐CT). Articles including information related to these research themes were searched for independently by H. S., Y. H., TF, S. O., and K. O. using PubMed and MEDLINE between January 2005 and July 2020. In PubMed, the search terms “esophageal cancer,” “gastric cancer,” “colorectal cancer,” and “TNM staging” were used. In MEDLINE, the search terms used in Cochrane Reviews were used (advanced search system, Appendix 1).^27^ The relevance of each article was evaluated (by H. S., Y. H., S. O., and T. F.) and categorized as either relevant or irrelevant. Irrelevant articles were excluded from the review.

### 2.2 Data extraction

Key messages and information were extracted from each article and organized. The following information from eligible articles was used: authors, title, countries of origin, publication year, total sample size, study design, study period, diagnostic modality, conclusion, and summary statistics (sensitivity, specificity, and number of positive and negative patients) for diagnosis. We focused on two statistical measurements of diagnostic accuracy of the modality: sensitivity (the proportion of positively diagnosed patients with disease) and specificity (the proportion of negatively diagnosed patients without disease).

## 3 RESULTS

### 3.1 Studies included in this paper

Our systematic search identified 23 126 articles using PubMed and MEDLINE. After a manual search of eligible papers, 3553 studies (640 esophageal cancer studies, 587 gastric cancer studies, and 2326 colorectal cancer studies) were considered eligible based on their title and abstract. After a full‐text search, a final set of 55 studies (17 esophageal cancer studies, 26 gastric cancer studies, and 12 colorectal cancer studies) were used to evaluate the accuracy of TNM staging.

### 3.2 Esophageal cancer staging

#### 3.2.1 Diagnosis for T4 invasion

**FIGURE 1:**
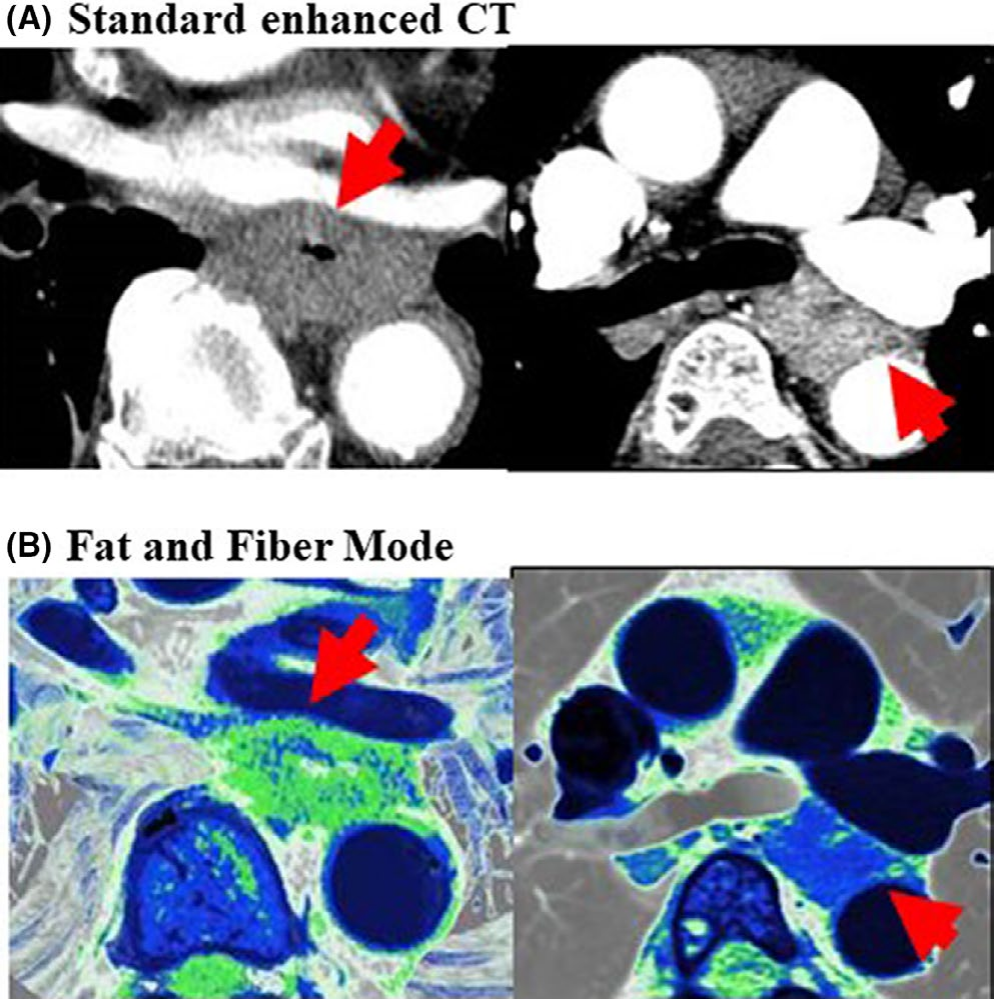
Representative CT images for clinical staging in esophageal cancer. The differential diagnosis between T4 and T3 tumors in esophageal cancer by fat and fiber mode. (A) Standard enhanced CT, (B) Fat and fiber mode

Computed tomography has been used for the majority of diagnostic modalities for T4 ever since Picus et al^28^ and Thompson et al^29^ first reported that CT images were useful to detect T4 invasion of esophageal cancer, with 80% accuracy. Recently, endoscopic ultrasonography (EUS) and MRI have also become standard tools to predict T4 invasion. Six recent studies in five reports^30^, ^31^, ^32^, ^33^, ^34^ were selected to evaluate the diagnostic impact of predicting T4 invasion of esophageal cancer (Table 1A). The sensitivity ranged from 27.3% to 84%, with 69% to 100% specificity. Although the accuracy of EUS was the highest among these diagnostic modalities, CT or MRI are still appropriate modalities in cases of stenosis or obstruction due to the tumor, which make EUS examination impossible. On the other hand, EUS or MRI was appropriate to determine non‐T4 status. The most reliable diagnostic modalities include a combination of EUS and CT. Kobayashi et al analyzed the characteristics of the esophageal motion and esophageal internal target volume margins to assess the differences between clinical T1‐T3 and clinical T4 using four‐dimensional CT.^35^ Although the accuracy of EUS was the highest among these diagnostic modalities, CT or MRI were appropriate modalities to detect T4 status. Figure 1 shows the differential diagnosis between T4 and T3 tumors of esophageal cancer after chemoradiation therapy using an image reconstruction method according to the CT value of the tissue histology of enhanced CT, so called fat and fiber mode.^36^ In this examination, the contrast agent (3 mL/kg body) was administrated and the legions were scanned with a 50‐second delay and a thickness of 1 mm. The fibrotic area induced by the chemoradiation therapy was emphasized as green and the presence of a fibrotic layer between the tumor and the adjacent organs could be interpreted as not T4.

**TABLE 1:** The summary of diagnostic modalities for TNM staging in esophageal cancer

Year	Author [Ref]	Country	Journal	Modality	Number of patients	Sensitivity (%)	Specificity (%)	Accuracy (%)
A) The summary of diagnostic accuracy for T4 in esophageal cancer.								
2013	O'Farrell NJ [30]	Ireland	World J Surg	EUS	222	66	93	71
2016	Lin‐na Luo [31]	China	Plos One	EUS	2880	84	96	79
2018	Jie Yang [32]	China	Ann Surg Oncol	EUS	1434	27	99	99
2018	Jinrong Qu [33]	China	Eur Radiol	EUS	43	57	100	68
2018	Jinrong Qu [33]	China	Eur Radiol	MRI	43	71	100	91
2018	Yue Zhou [34]	China	World J Gastroenterol	CT	120	84	69	72
B) Summary of diagnostic accuracy for N staging in esophageal cancer.								
2008	van Vliet [37]	The Netherlands	Br J Cancer	EUS	1841	80	70	75
2008	van Vliet [37]	The Netherlands	Br J Cancer	CT	943	50	83	63
2008	van Vliet [37]	The Netherlands	Br J Cancer	PET	424	57	85	67
2012	Li H [38]	China	Eur Radiology.	CT	205	76	75	76
2012	Yano M [39]	Japan	Esophagus	PET/CT	81	32	70	53
2014	Yamada H [40]	Japan	Surgery Today	PET	258	26	98	82
2016	Parry K [41]	The Netherlands	Eur J Surgical Oncology	EUS + CT	266	31	84	68
2017	Foley KG [42]	UK	Clin Radiol.	PET	112	40	77	55
2018	Jeong DY [43]	Korea	Cancer Medicine	EUS	435	90	42	75
2018	Jeong DY [43]	Korea	Cancer Medicine	PET/CT	435	89	39	73
2018	Jiancheng li [44]	China	Rev Assoc MeD Bras	CT	305	55	88	82
2018	Harrington C [45]	United Kingdom	World J Gastrointest Endosc.	PET/CT	121	93	50	59
2018	Liu J [46]	China	Eur Radiol.	CT	204	67	92	87
C) Summary of diagnostic accuracy for M staging in esophageal cancer.								
2004	van Westreenen [47]	The Netherlands	J Clin Oncol.	PET	452	67	97	86
2008	van Vliet [37]	The Netherlands	Br J Cancer	CT	437	52	91	77
2008	van Vliet [37]	The Netherlands	Br J Cancer	PET	475	71	93	85
2018	Lucas Goense [48]	USA	Eur J Nuc Med Mol Imag	PET	783	75	94	92

#### 3.2.2 Lymph node staging

A total of 13 studies in 10 manuscripts^37^, ^38^, ^39^, ^40^, ^41^, ^42^, ^43^, ^44^, ^45^, ^46^ evaluated the diagnostic impact of EUS/CT/PET on the nodal involvement in esophageal cancer (Table 1B). Four out of 13 eligible studies used combination diagnosis with either PET/CT or EUC/CT. The sensitivity of nodal involvement ranged from 29% to 94%, and the specificity ranged from 38% to 98% (Table 1B).

#### 3.2.3 Diagnosis for distant metastases

Four studies in three manuscripts^37^, ^47^, ^48^ evaluated the diagnostic accuracy of imaging to detect distant metastases (Table 1C). The useful modalities were CT and PET. The advantage of CT was its high resolution to detect the lesion with an accuracy of 77%,^37^ while the advantage of PET was the ability to perform whole‐body scanning with highly qualitative contrasted metastatic lesions identified by high glucose uptake with an accuracy of 85% ~ 92%.^46^, ^47^, ^48^, ^49^, ^50^, ^51^ These accuracies were around 10% greater than that of CT; therefore, CT and PET should be used together as morphological and qualitative modalities.

#### 3.2.4 Algorithm of image modalities for clinical staging in esophageal cancer

**FIGURE 2:**
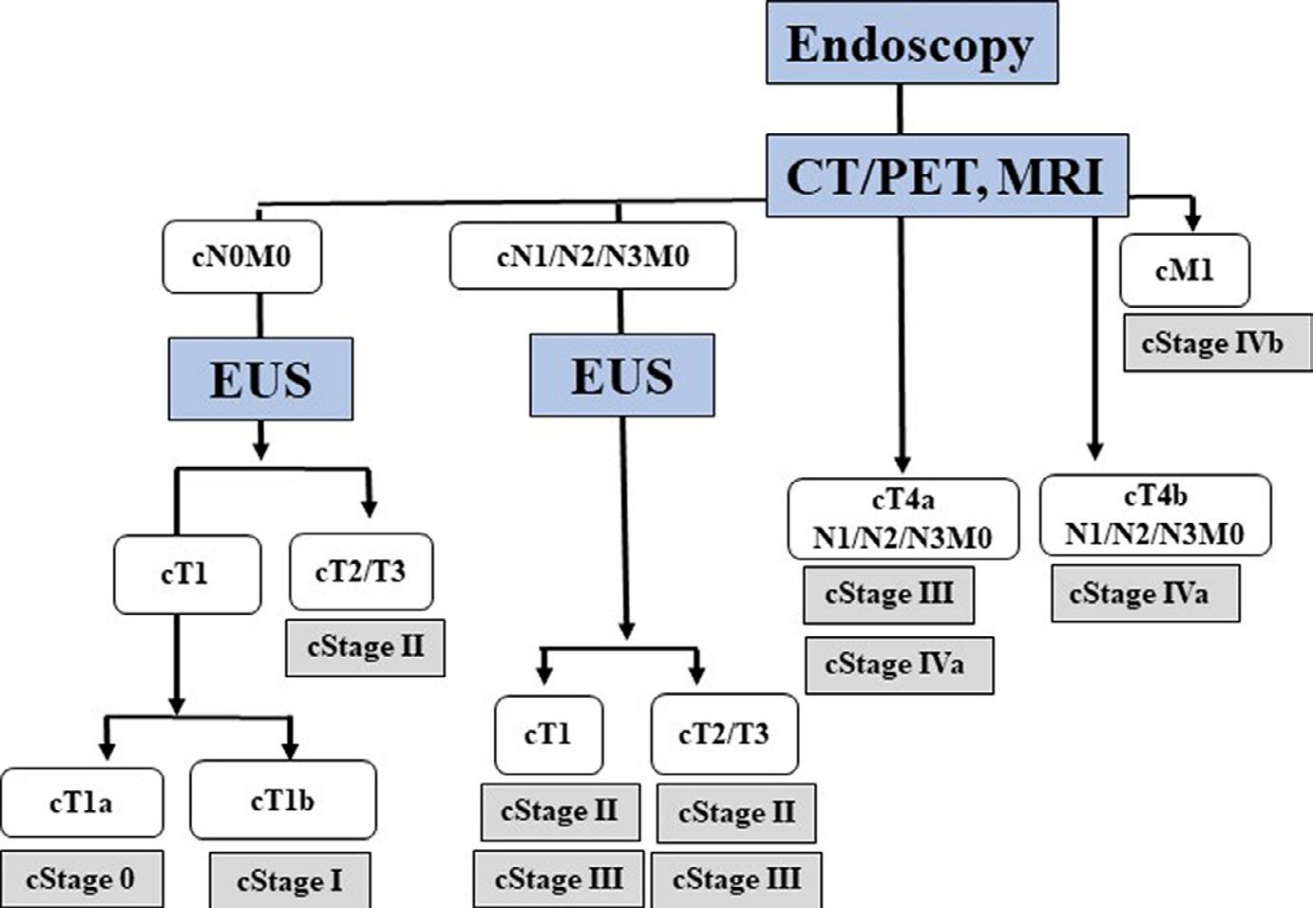
Algorithm of diagnostic modalities for clinical staging in esophageal cancer

Based on these findings, we suggest an algorithm of image modalities for clinical staging in esophageal cancer (Figure 2). After routine endoscopic examination to determine the pathology by biopsy and exclude T1 tumors, PET‐CT and/or MRI should be performed. Surgical resection should be performed in patients without distant metastasis or T4 invasion, whereas PET‐CT should be employed to detect further metastasis in patients with distant metastasis. Chemotherapy with or without surgery or radiation should be selected depending on the involved lesions. Precise assessment by EUS should be performed to select tumors indicated for endoscopic resection when the tumor depth is evaluated as T1. Findlay et al reported “pragmatic staging” of esophageal cancer using decision theory involving selective EUS, PET, and laparoscopy.^51^ They concluded that EUS was used in 71.8% of patients with T2‐T4a disease and that it was moderately accurate for pT1 N0 disease. PET‐CT altered management in 23.0% of patients and laparoscopy in 7.1% of patients, including those with T2 and distal esophageal tumors. Furthermore, although EUS provided additional information on T and N categories, its risk outweighed any potential benefits in patients with T2‐T4a disease on CT.

### 3.3 Gastric cancer staging

#### 3.3.1 T staging

T staging of gastric cancer is evaluated by conventional endoscopy, EUS, CT, and MRI. A total of 10 studies in eight manuscripts^21^, ^52^, ^53^, ^54^, ^55^, ^56^, ^57^, ^58^ evaluated the diagnostic accuracy for T staging (Table 2A). In East Asia, where early gastric cancer is frequently detected, conventional endoscopy is the main modality of T staging. EUS could provide additional diagnostic value for distinction between T1a (m) and T1b (sm). Hwang et al concluded that the accuracy of multidetector‐row CT was close to that of EUS and both EUS and multidetector‐row CT were useful complementary modalities for the locoregional staging of gastric cancer.^56^ For advanced gastric cancer, T staging can be performed by CT, and has reported diagnostic accuracies of 82%,^53^ 77%,^54^ 91%,^57^ 80%,^59^ 78%,^58^ and 73%.^21^ The reported accuracies from retrospective studies involve potential bias about patient selection. The diagnostic accuracy is likely to be better when higher numbers of T1 cases are included in studies. Fukagawa reported a diagnostic accuracy of 73% in a large‐scale prospective study limited to advanced tumor. The diagnostic ability of MRI for T staging is reported to be 78%,^55^ which is similar to that of CT.

**TABLE 2:** The summary of diagnostic accuracy for TNM staging in gastric cancer

Year	Author [Ref]	Location	Modality	T	Number of Patients	Sensitivity (%)	Specificity (%)	Accuracy (%)
A) Summary of diagnostic accuracy for T staging in gastric cancer.								
2008	Puli [52]	USA	EUS	T1‐4	1896	88/82/90/99	60	NA
2009	Pan [53]	China	CT	T3	135	85	81	82
2010	Hwang [54]	Korea	EUS	T1‐4	277	NA	NA	75
2010	Hwang [54]	Korea	CT	T1‐4	277	NA	NA	77
2010	Huang [55]	China	MRI	T1/2 vs. T3/4	213	93	91	NA
2011	Choi [56]	Korea	EUS	T1	955	NA	NA	67
2011	Makino [57]	Japan	CT	T1‐4	616	NA	NA	91
2013	Feng [58]	China	EUS	T1‐4	610	NA	NA	77
2013	Feng [58]	China	CT	T1‐4	610	NA	NA	78
2017	Fukagawa [21]	Japan	CT	T2 /T3,4	1222	85	49	73
B) Summary of diagnostic accuracy for N staging in gastric cancer.								
2007	Bentrem [60]	US	EUS	T1‐4(T1:30%)	223	75	66	71
2009	Ahn [61]	Korea	CT	T1	434	17	90	84
			EUS	T1	71	17	97	90
2010	Pan [62]	China	CT	T1‐4	350	NA	NA	87
2012	Seevaratnam [63]	Canada	CT	T1‐4	2909	77	78	66
2012	Seevaratnam [63]	Canada	MRI	T1‐4	109	85	75	53
2012	Seevaratnam [63]	Canada	PET	T1‐4	422	40	60	98
2013	Feng [58]	China	EUS	T1‐4	610	NA	NA	49
2013	Feng [58]	China	CT	T1‐4	610	NA	NA	45
2013	Hasegawa [64]	Japan	CT	T1‐4(T1:60%)	315	46	97	81
2014	Fujikawa [65]	Japan	CT	T1	761	4.3	99	90
2015	Wang [66]	China	CT	T1‐4	6788	67	84	NA
2015	Mocellin [67]	Italy	EUS	T1‐4	3573	83	67	NA
2017	Fukagawa [21]	Japan	CT	T2‐4	1241	63	66	64

Year	Author	Location	Number of Patients	Indication of SL	Yield	False negative (%)
C) Summary of diagnostic accuracy for peritoneal metastases in gastric cancer by staging laparoscopy.						
2006	Sarela [70]	US	657	Resectable GC & EGJ	23	10% (41/401) (p:56%)
2013	Munasinghe [71]	UK	316	Resectable GC & EGJ	23	0% (0/183)
2014	Ishigami [72]	Japan	178	GC ≧T2	43	NA
2015	Convie [73]	UK	295	Resectable GC, EGC & EC	21	NA
2015	Mirza [74]	UK	378	Resectable GC, EGC & EC, ≧T3 or N+	14	NA
					13	
2016	Simon [75]	France	116	Resectable GC, EGC & EC, ≧T3 or N+	13	NA
2016	Ikoma [76]	US	711	Resectable GC & EGC (EGJ: 43.2%)	36	NA
2016	Hu [77]	China	582	GC ≧T2	26	NA
2017	Hosogi [78]	Japan	120	≧5 cm and/or bulky N	45	5.9% (1/17)
2018	Irino [79]	Japan	156	Large type 3 & type 4, bulky N/PAN, suspicious for P	47	11% (7/66)

#### 3.3.2 Lymph node staging

The clinical evaluation of lymph node metastases in gastric cancer is performed by either CT or EUS. A total of 13 studies in 10 manuscripts^21^, ^57^, ^58^, ^59^, ^60^, ^61^, ^62^, ^63^, ^64^ evaluated the diagnostic accuracy for N staging (Table 2B). Pathological N staging is determined by the number of positive nodes. However, accurate diagnosis of the number of positive nodes is challenging, and positive/negative is incorporated into the clinical staging (TNM 8th). In a review that included a high volume of cases of stage T1‐T4 cancer, the sensitivities and specificities were reported as 83% and 67%, respectively, by Mocelin et al,^65^ 77% and 78%, respectively, by Seevaratnam et al,^63^ and 67% and 84%, respectively, by Wang et al.^66^ The incidence of lymph node metastases was higher in advanced tumors than in early tumors. When limited to T1 tumors, the sensitivities and specificities were reported to be 17% and 90%, respectively, by Ahn et al^58^ and 4.3% and 98%, respectively, by Fujikawa et al.^67^ When limited to T2‐T4, the specificity and sensitivity were reported to be 63% and 66%, respectively, by Fukagawa et al.^21^

**FIGURE 3:**
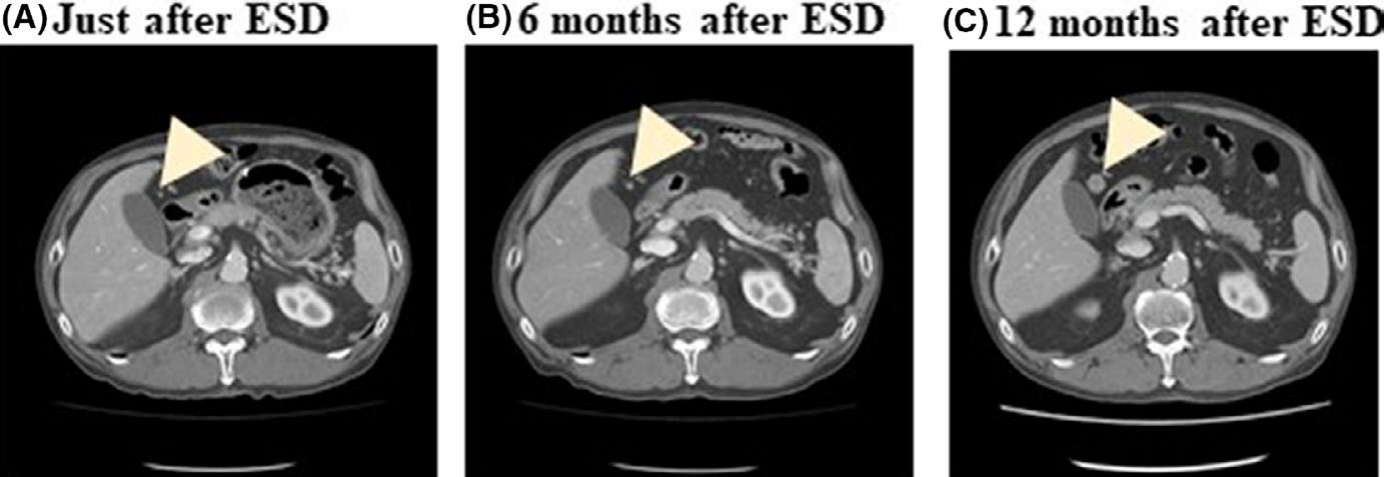
Representative CT images for clinical staging in gastric cancer. Diagnostic difficulties for small lymph node metastases in gastric cancer. (A) Just after ESD, no lymph node swelling. (B) 6 mo after ESD, no lymph node swelling. (C) 12 mo after ESD, 1.2 cm sized lymph node swelling is shown nearby gallbladder. This lymph node is clinically metastatic at the No. 6 station

One of the reasons for difficulties in lymph node diagnosis is the diagnostic difficulty for small‐sized lymph node metastases. Figure 3 shows a series of CT images at the same position after endoscopic submucosal dissection (ESD) in a patient who underwent ESD for T1a early gastric cancer. The pathological depth of the resected specimen was sm2, and there was the possibility of simultaneous lymph node metastases. However, this patient chose to be monitored using CT examinations every 6 months without additional surgery for lymph node dissection. The lymph node was found to be clinically metastatic at the No. 6 station. Following this CT finding, the patient underwent distal gastrectomy and one lymph node was found to be pathologically positive for metastasis at the No. 6 station, which was compatible with the CT findings. Looking at these CT images, a tiny lymph node was visible (Figure 3A,B) in the same area, with swollen node visible (Figure 3C). This tiny lymph node may have been positive for metastasis at that time but was not found to be clinically positive due to its small size. This patient underwent distal gastrectomy after this CT finding, and one lymph node was pathologically positive for metastasis at No. 6, the same with the CT finding. Looking back at these CT images, a tiny lymph node was visible (in A and B) at the same area with swollen node in (C). This tiny lymph node was positive for metastasis at that time, which was not clinically positive for its small size.

#### 3.3.3 Diagnosis for distant metastases

Peritoneal dissemination is diagnosed using CT, with findings of ascites and multiple mesenteric or omental nodules; however, its diagnostic accuracy is not high (Table 2C).^68^ The standard therapeutic strategy for advanced gastric cancer with peritoneal dissemination is systemic chemotherapy without gastrectomy, as determined by the REGATTA trial.^69^ The detection of small peritoneal dissemination by staging laparoscopy can avoid unnecessary laparotomy. A total of 10 manuscripts^70^, ^71^, ^72^, ^73^, ^74^, ^75^, ^76^, ^77^, ^78^, ^79^ evaluated the diagnostic accuracy of peritoneal metastases by staging laparoscopy (Table 2C). The detection ratio of peritoneal dissemination was found to be 7.8%–36%.^77^ In Western countries, the indication of staging laparoscopy is basically resectable advanced gastric cancer diagnosed as P0 by routine examination modality as CT, ultrasound, and EUS. In contrast, 46%–53.4% was reported in Japan^72^, ^76^, ^78^, ^79^ because staging laparoscopy is performed for more limited patients who are potentially associated with peritoneal dissemination, including type 4, large‐sized type 3 (> 8 cm), and high lymph node metastases. The diagnostic accuracy of peritoneal dissemination by staging laparoscopy is not always 100%. The percentage of “false negatives” is reported to be 11%–17% in Japan and 0%–8% in Western countries.^80^ The reason for this discrepancy is considered to be the difference in the indication of staging laparoscopy.

#### 3.3.4 Algorithm of image modalities for clinical staging in gastric cancer

**FIGURE 4:**
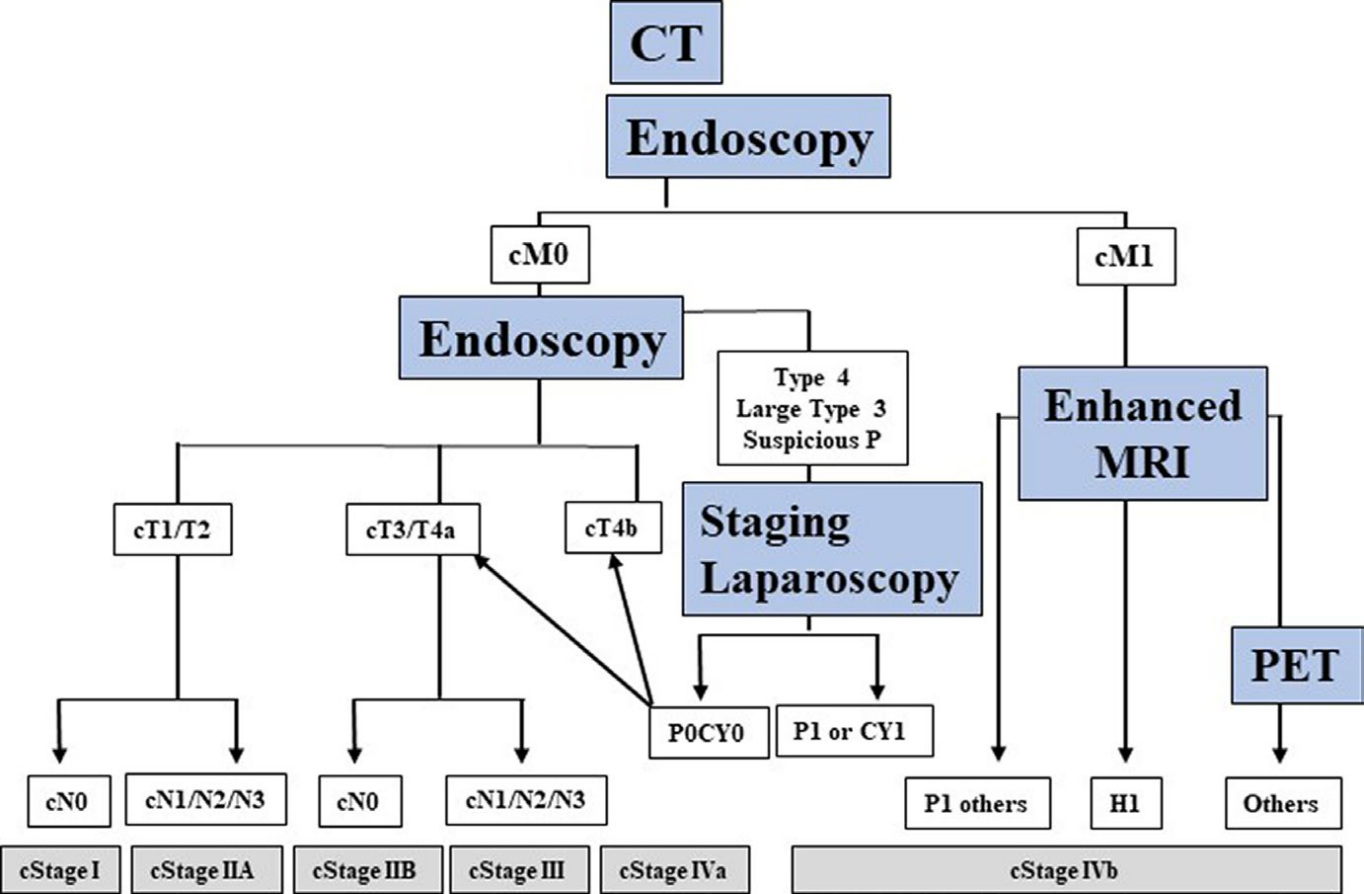
Algorithm of diagnostic modalities for clinical staging in gastric cancer

Figure 4 shows an algorithm of image modalities for clinical staging in gastric cancer. Endoscopy and CT scan should be performed first for pretreatment diagnosis. If there are no findings for distant metastases (cM0) by CT scan, clinical stage is defined by T staging and N staging. In cases of type 4 and large type 3 tumors, staging laparoscopy is recommended for screening peritoneal dissemination and positive cytology that cannot be detected by CT. If distant metastases are diagnosed by CT (cM1), the patient is evaluated as cStage IVb. For liver metastases, enhanced MRI is effective for detecting small metastatic nodules; therefore, correct diagnosis of the number of liver metastases is available.^81^ Distant metastases of other sites (including lung, bone, adrenal gland, distant lymph node) should be confirmed by PET.

### 3.4 Colorectal cancer staging

#### 3.4.1 T staging

The selected papers are summarized in Table 3.^82^, ^83^, ^84^, ^85^, ^86^, ^87^, ^88^, ^89^, ^90^, ^91^, ^92^, ^93^ For T staging (Table 3A), Chen et al revealed that dual‐energy CT showed a high accuracy, with a sensitivity of 90% and specificity of 97%.^83^ Komono et al^86^ advocated the new criteria using CT–colonography with multiplanar reconstruction to differentiate between T2, T3, and T4a. They focused on new blood vessels produced by tumor angiogenesis at the subserosal layer, designated as “bordering vessels.” They defined the criteria that tumors that do not involve bordering vessels and have a smooth outer border are considered T2, while those with a rough border are T3 and those that involve bordering vessels are T4. Using these new criteria, we demonstrated that the accuracy of diagnosing T3 was 81% and that of T4a was 97%. In rectal cancer, MRI is considered the gold standard for preoperative T staging.^94^

**TABLE 3:** The summary of diagnostic modalities for TNM staging in colorectal cancer

Year	Author [Ref]	Country	Journal	Modality	Number of patients	Sensitivity (%)	Specificity (%)	Accuracy (%)
A) The summary of diagnostic modalities for T staging in colorectal cancer.								
2014	Cho SH [82]	Korea	Am J Roentgenol	MRI	146	74	87	85
2014	Chen CY [83]	China	PLos One	CT	103	90	97	95
2017	So JS [84]	Korea	Ann Coloproctol	CT	285	90	68	55
2018	Malmstrøm ML [85]	Denmark	Int J Colorectal Dis	CT	615	65	89	49
2019	Komono A [86]	Japan	Int J Colorectal Dis	CT	172	79	99	97
2019	Korsbakke K [87]	Sweden	Acta Radiologica Open	CT	383	28	93	74
B) Summary of diagnostic accuracy for N staging in colorectal cancer.								
2014	de Vries FE [88]	Netherlands	Eur J Surg Oncol	CT	106	71	41	54
2016	Ogawa S [89]	Japan	Ann Surg Oncol	MRI	449	73	55	64
2017	So JS [84]	Korea	Ann Coloproctol	CT	285	72	63	55
2017	Lee JY [90]	Korea	Intest Res	PET	220	44	84	67
2017	Lee JY [90]	Korea	Intest Res	CT	220	59	65	62
2019	Korsbakke K [87]	Sweden	Acta Radiologica Open	CT	383	55	66	61
C) Summary of diagnostic accuracy for M staging in colorectal cancer.								
2016	Oh JW [92]	Korea	Biomed Res Int	PET/CT	67	95	100	97
2016	Oh JW [92]	Korea	Biomed Res Int	Gd‐MRI	67	98	93	98
2016	Colagrande S [93]	Italy	Eur J Radiol	MRI	115	97	85	96
2017	Lee JY [90]	Korea	Intest Res	PET	220	79	94	93
2017	Lee JY [90]	Korea	Intest Res	CT	220	79	87	86

#### 3.4.2 Lymph node staging

For lymph node staging (Table 3B), the sensitivity was found to range from 44% to 73%, and the specificity ranged from 41% to 84%.^84^, ^85^, ^86^, ^87^, ^88^, ^89^, ^90^ The accuracies of these studies were around 50%–60%. PET showed a relatively high specificity of 84%, but a sensitivity of only 44%.^91^ These results highlight the requirement for more reliable modalities. Colon cancer patients were surgically resected regardless of preoperative nodal status, and thus clinical N staging is not essential in these patients. Neoadjuvant therapy is only considered for locally advanced colon cancer.^87^ For rectal cancer, neoadjuvant chemoradiation therapy is more common against nodal positive cancer in Western countries. Nonetheless, the accuracy of clinical staging has been reported to be medium.^94^

#### 3.4.3 Diagnosis for distant metastases

**FIGURE 5:**
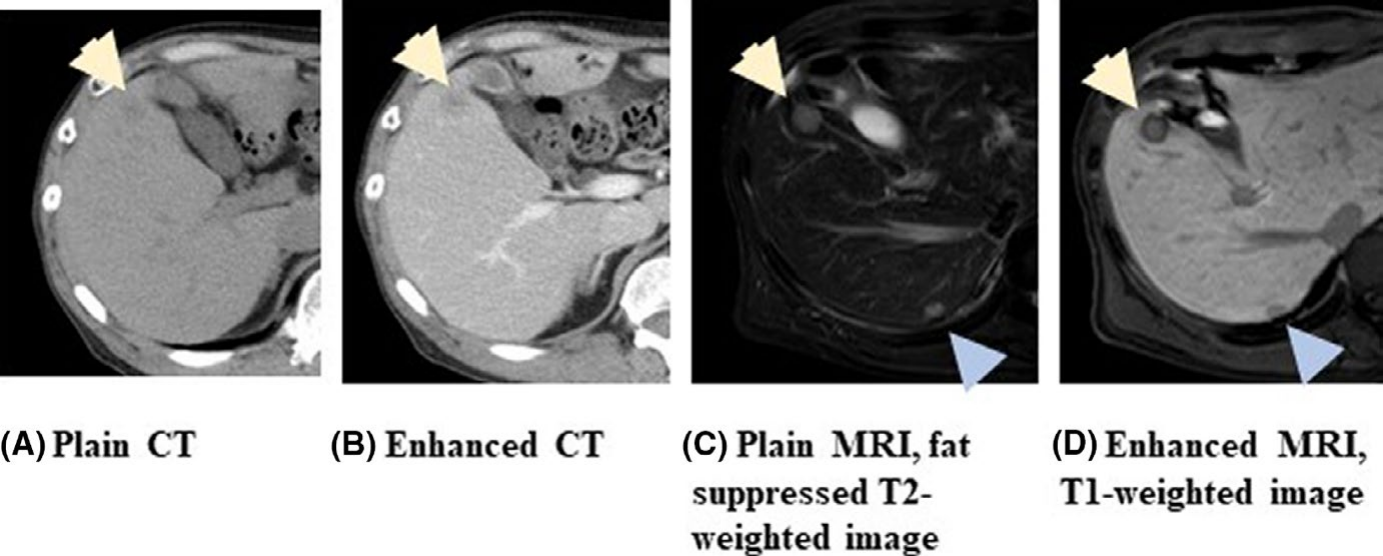
Representative CT images and MRI images to detect liver metastasis in the patients with colorectal cancer. (A) Plain CT, (B) Enhanced CT, (C) Palin MRI, fat suppressed T2‐weighted image, (D) Enhanced MRI, T1‐weighted image

The diagnostic accuracy of imaging to detect distant metastases is shown in Table 3C. Oh et al compared the use of MRI and PET‐CT to detect liver metastasis.^92^ The per patient analysis revealed similar specificities and sensitivities between the modalities. On the other hand, the per nodule analysis showed that the sensitivity of PET‐CT was 68.7%, which was significantly lower than that of MRI (96.2%). Colagrande et al and Moreno et al also demonstrated a high accuracy of MRI to detect liver metastasis.^94^, ^95^ Figure 5 illustrates the superiority of MRI to detect small liver metastasis over enhanced CT. CT could only detect a 1‐cm sized lesion in the S5 segment, whereas MRI was able to detect the lesions as well as another 4‐mm sized lesion on the back side. A comparison of CT and MRI to detect liver metastasis in colorectal cancer was conducted. MRI was able to detect small metastasis of the liver that could not be detected by CT. Georgakopoulos et al revealed that PET‐CT was able to detect extrahepatic disease, which was missed by conventional imaging in 50% of patients who were found to have liver metastasis prior to surgey.^95^ These findings may alter the treatment strategy.

#### 3.4.4 Algorithm of image modalities for clinical staging in colorectal cancer

**FIGURE 6:**
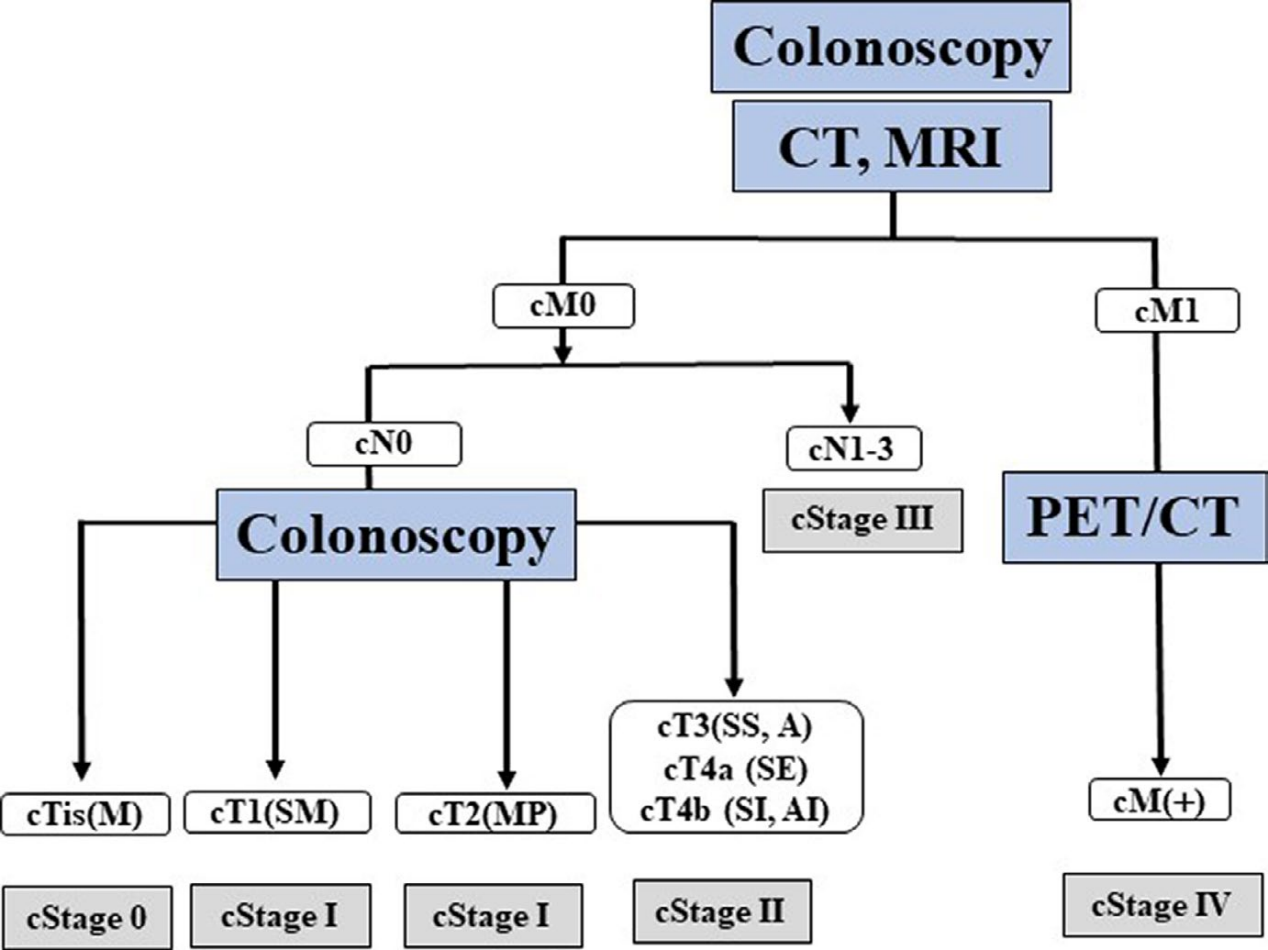
Algorithm of diagnostic modalities for clinical staging in colorectal cancer

Based on these results, we propose an algorithm of image modalities for clinical staging in colorectal cancer (Figure 6). Colonoscopy, CT, and MRI should preoperatively be performed in colorectal cancer patients. CT–colonography is useful for T staging, and MRI is much more sensitive than CT for the detection of small liver metastasis. Surgical resection should be performed in patients without distant metastasis, whereas PET‐CT should be used to detect extrahepatic metastasis in patients with distant metastasis. Chemotherapy with or without surgery or radiation should be selected according to the involved lesions.

A comparison of the best diagnostic accuracy for clinical N staging among esophageal, gastric, and colorectal cancers are shown in Table 4. The common criteria for metastatic nodes were “round shape” and “enhancement” in gastrointestinal cancers. The optimal cutoff size to classify the positive lymph nodes differed according to the type of cancer as follows: 5‐10 mm in esophageal nodes,^96^ 8‐10 mm in gastric nodes,^21^ and 4‐5 mm in colorectal nodes.^88^, ^97^ Although the diagnostic accuracy in esophageal cancer was relatively higher than that in gastric and colorectal cancers, the accuracies in all three types of cancer were unsatisfactory.

**TABLE 4:** Comparison of the best diagnostic accuracy for clinical N staging between esophageal, gastric, and colorectal cancers

Reference cutoff size	Esophagus [96] 5 mm	Gastric [21] 8 mm	Colorectal [97] 4 ~ 5 mm
Sensitivity	67%	63%	73%
Specificity	92%	66%	55%
Accuracy	87%	64%	64%

## 4 DISCUSSION

This systematic review of clinical staging of gastrointestinal cancers included 55 articles published between January 2005 and July 2020 that were retrieved from PubMed/MEDLINE. Since the present review examined patient selection for neoadjuvant therapy, the main targets of diagnosis were T2‐T4, positive nodes, and distant metastases. Although several systematic reviews have evaluated the performance of clinical staging for gastrointestinal cancers, most focused on just one cancer type. The present review evaluated the diagnostic modalities to detect T2‐T4 invasion, nodal involvement, and distant metastases in patients with gastrointestinal cancers based on the studies published during the same period. Favorable diagnostic modality for lymph node metastasis in each type of cancer differed: however, the sensitivities ranged from 60% to 80%. PET–CT was the best modality to detect distant metastases for esophageal cancer, staging laparoscopy was the best modality for detecting peritoneal metastasis of gastric cancer, and MRI was the best modality for detecting liver metastasis of gastric cancer and colorectal cancer.

Detection of lymph node metastases in superficial gastric cancer is to differentiate tumors for ESD indication. Since superficial esophageal cancer is more likely have lymph node metastases than other gastrointestinal cancers,^98^ precise evaluation of lymph node metastases is essential to determine the indication for ESD. These topics are reviewed elsewhere.^99^, ^100^

Detection of T4 invasion and distant metastases are the most important critical issues regarding the clinical staging of advanced esophageal cancer. Based on the present systematic review, CT was found to be the best modality to evaluate potential invasion to adjacent organ, while PET‐CT or EUS/CT was useful to detect nodal metastases. Since subtotal esophagectomy is one of the most stressful surgical procedures, neither T4 invasion or distant metastases should be detected prior to surgery to differentiate noncurative tumors. Based on selected papers, although the sensitivities for the detection of T4 invasion were not high enough, the specificities were nearly 100%. The majority of suspected T4 cases were treated by chemoradiation rather than surgery; therefore, the number of patients included in the papers were limited. The positive predictive value for distant metastases gradually increased according to the time period of published papers; however, the sensitivities and accuracies remained unsatisfactory, although the resolution of PET images has improved during the last 10 years.^101^ The identification of patients who may not benefit from potentially curative surgery may be associated with high resolution. However, the present review also demonstrated that the use of PET‐CT restaging resulted in a 5% false positive rate, which may introduce unnecessary physical and psychological intervention to the patient via additional testing and anxiety.

Clinical staging after chemoradiation therapy should be essential for esophageal cancer. Among previous reports using various diagnostic modalities, PET‐ CT may be the best tool for response assessment after neoadjuvant chemoradiotherapy. Stiekema et al reported that maximum standardized uptake value, metabolic tumor volume, and total lesion glycolysis were correlated with the pathologic response.^102^ Assessment of changes to these parameters may be the best tools for restaging after neoadjuvant therapy.

Important critical issues of clinical staging of gastric cancer include detection of early gastric cancer for ESD indication, T3 invasion and more with lymph node metastases positive for the indication of NAC, and distant metastases. The detailed indication of ESD is defined in the Japanese Gastric Cancer Association guidelines. Mucosal cancer is a basic target for ESD, and clinical distinction between mucosal and submucosal invasion by endoscopic examination is required. The positive predictive value for pT1b (sm) by endoscopic diagnosis was reported to be 63%–89%,^56^, ^103^, ^104^, ^105^ and additional diagnostic values by EUS were not demonstrated in some reports.^103^, ^106^ The diagnostic characteristics of submucosal invasion are not described clearly and diagnostic ESD is performed in some cases. In Western countries, the standard therapeutic strategy for advanced gastric cancer is NAC based on the results of pivotal clinical trials, such as the FLOT trial^20^ and others.^16^, ^17^, ^18^

Surgical outcomes of p stage I/II gastric cancer patients are favorable, and p stage III patients are the main target of NAC. However, p stage I/II patents were included in the NAC group in the FLOT trial due to clinical misdiagnosis. In JCOG1302A,^23^ the proportion of p stage I patients who were diagnosed as clinical stage III, T3/T4 and N1‐3, and T3/T4 were 4.6%, 6.5%, and 12%, respectively. The sensitivities for p stage III patients were 52%, 65%, and 88%, respectively. Based on these findings, the eligibility criteria in JCOG1509 regarding NAC for advanced gastric cancer is defined as “T3/4 and N1‐3.” An essential consideration of clinical diagnosis of gastric cancer is “How can an accurate diagnosis of T3/4 and N positive be performed?” Difficulties remain concerning the accurate diagnosis of lymph node metastases of gastric cancer patients because lymph node evaluation by size alone has potential limitations.^107^ A cutoff value of 8 mm is commonly used, but smaller‐sized lymph node metastases are frequently seen, especially for poorly differentiated adenocarcinoma. If a smaller cutoff value is defined for metastases, diagnostic “false positives” will be more frequent. Even a diagnosis of node positive/negative is not sufficiently accurate that we can give up the clinical N staging based on the number of metastatic nodes. The clinical diagnosis of peritoneal dissemination is commonly determined by ascites, thickness of omentum, hydronephrosis, and definite disseminated nodules by CT imaging, but small disseminated nodules cannot be detected by imaging. Staging laparoscopy is recommended prior to surgery for advanced gastric cancer patients with possible peritoneal dissemination (linitis plastica, large‐sized tumor, and suspicious findings of dissemination by imaging).

In colorectal cancer, the important factors to consider when selecting candidates for neoadjuvant therapy are tumor depth and distant metastases. In stage II/III, neoadjuvant therapy is uncommon and upfront surgery is the first priority in colorectal cancer.^91^ Therefore, these findings indicate that N staging is not important. One of the most important clinical features is liver metastases in colorectal cancer. Therefore, MRI should first be performed prior to surgery, which is reported to be better in detecting liver metastasis than PET‐CT and CT.^92^, ^93^, ^108^ Afterwards, CT should be performed to detect lung metastases as well as for T staging. Regarding the diagnostic definition of lymph node metastases in colorectal cancer, Ogawa et al reported a better diagnostic accuracy using a cutoff size of 5 mm compared with 10 mm.^89^ These cutoff values are relatively smaller than those for gastric cancer.

At present, the number of positive nodes cannot be diagnosed accurately by imaging. Since the tumor depth is significantly associated with the number of positive nodes, combination diagnosis using tumor depth and clinical positive nodes may be the most reliable clinical diagnosis under the current performance of imaging technology. On the other hand, the accuracy of diagnostic imaging to detect distant metastases from gastrointestinal cancers is becoming more reliable with the use of PET‐CT and/or MRI with the latest technologies. So far, we speculated that PET was useful for the esophagus squamous cell carcinoma, but less useful for gastric and colorectal adenocarcinomas. Highly antigenic tumors generally tend to develop swelling of metastatic lymph nodes, whereas low antigenic tumors tend to have smaller metastatic lymph nodes.

The rate of accurate diagnosis of conventional diagnostic imaging was evaluated in patients who underwent radical surgery without preoperative treatment. However, many advanced cancers will become candidates for preoperative treatment. Therefore, it will be necessary to perform diagnostic imaging before and after preoperative chemotherapy to monitor changes in staging and the rate of agreement with postoperative pathological staging. It is not possible to verify whether pretreatment staging was correct in patients undergoing preoperative chemotherapy. However, if the staging by diagnostic imaging after preoperative treatment matches the postoperative pathological staging, it may be possible to ensure the accuracy of the staging prior to treatment. In the future, more accurate pathological therapeutic effects and staging will be required after preoperative treatment. In patients receiving preoperative treatment, difficulties remain in terms of lymph node metastasis diagnosis and the usefulness of PET is predicted to become more important.

In conclusion, our literature review suggests that the recent diagnostic modalities can make precise differential diagnoses for T4, N1, and M1 for gastrointestinal cancers. However, the accuracy is still not sufficient to design preoperative treatment strategies. The most important purpose of clinical staging is to determine whether neoadjuvant therapy should be performed on each patient. Overstaging could occur in some patients without a standard algorithm for clinical staging and may lead to overtreatment. Accurate diagnostic modalities that adhere to a standard algorithm may improve both oncological outcomes and patient quality of life. Since there are only a few large‐scale prospective cohort studies in this field, further multi‐institutional prospective studies are required.

## DISCLOSURE

Funding: This work has been partly supported by a research grant of Toho University School of Medicine.

Conflict of Interest: The authors have no conflict of interest to declare.

## APPENDIX 1

### MEDLINE SEARCH TERMS

1. exp Tomography, Emission‐Computed/
2. exp Tomography, X‐Ray Computed/
3. exp Magnetic Resonance Imaging/
4. (computed tomography* or CT or CECT or MDCT or MSCT or magnetic resonance imaging or MRI or emission tomography or PET). mp. [mp = title, abstract, original title, name of substance word, subject heading word, keyword heading word, protocol supplementary concept word, rare disease supplementary concept word, unique identifier]
5. exp Ultrasonography/
6. (ultrasound or ultrasonography* or US or CEUS) .mp. [mp = title, abstract, original title, name of substance word, subject heading word, keyword heading word, protocol supplementary concept word, rare disease supplementary concept word, unique identifier]
7. 1 or 2 or 3 or 4
8. 5 or 6
9. exp Colorectal Neoplasms/
10. exp Stomach Neoplasms/
11. exp esophageal neoplasms/
12. 9 or 11 or 12
13. 8 and 12
14. limit 13 to yr="2005 ‐Current"
